# Correlation of Anticancer Drug Prices with Outcomes of Overall Survival and Progression-Free Survival in Clinical Trials in Japan

**DOI:** 10.3390/curroncol30020137

**Published:** 2023-02-01

**Authors:** Ayano Okabe, Haruto Hayashi, Hideki Maeda

**Affiliations:** Department of Regulatory Science, Faculty of Pharmacy, Meiji Pharmaceutical University, Tokyo 204-8588, Japan

**Keywords:** oncology, anticancer drugs, drug price, cost effectiveness, endpoint

## Abstract

Drug pricing methods vary extensively across countries. Japan calculates drug prices using cost accounting and based on the efficacy of similar drugs. This study investigated the relationship between drug prices and their clinical efficacy and usefulness using public information on anticancer drugs reimbursed by the National Health Insurance price listing between January 2009 and March 2020. We investigated drug characteristics, prices, and clinical benefits based on overall survival (OS) and progression-free survival (PFS). Eighty anticancer drugs were approved in Japan during the study period. The largest number (28 drugs, 35.0%) was approved based on PFS, 18 (22.5%) were approved based on OS, and 13 (16.3%) based on the response rate. The mean (±SD) drug price was JPY 88,416.2 (±148,974.7), while the median drug price (with quartiles) was JPY 21,694 (JPY 4855.0–JPY 93,396.8). Drug prices were significantly higher for PFS than for OS, while cost index—the drug price to extend PFS or OS by one day—did not differ significantly between PFS and OS. The relationship between the 46 drugs approved based on OS or PFS and their prices was examined. A correlation was found between drug prices and their clinical usefulness in terms of OS but not PFS.

## 1. Introduction

In recent years, high drug prices have been recognized as a societal problem in developed countries, including Japan [1]. Owing to Japan’s “super-aged” society, the proportion of its national budget allocated to healthcare is increasing steadily [2,3]. In Japan and other developed countries, cancer is a major cause of death. More than half of Japanese people are diagnosed with cancer in their lifetime, and the number of deaths due to cancer has been increasing [4].

As cancer treatment remains unsatisfactory for numerous types of cancer and patient categories, there is considerable demand for innovative, new anticancer drugs. This is especially true in cases of rare cancer types; novel anticancer drugs are often considered orphan therapy, which drastically increases their prices [5].

In addition to the true endpoint, overall survival (OS) surrogate endpoints—such as progression-free survival (PFS) and response rate (RR)—are considered primary endpoints for approving anticancer drugs, and clinical trial results are used to support such approval [6]. Although OS is the definitive hard endpoint of treatment, performing clinical trials with OS as the endpoint presents difficulties, such as the need for large-scale trials that span multiple years [7,8,9]. Thus, conducting oncological clinical trials based on surrogate endpoints has numerous benefits; the inclusion of surrogates and shorter endpoints would ensure faster oncological clinical trials and, therefore, quicker introduction of novel therapies for cancer patients [10].

Specifically, the extension of OS reflects the actual clinical benefit to cancer patients. However, prolonged PFS is used as a primary endpoint in Phase III trials. PFS is a surrogate endpoint under all circumstances, and it is not necessarily limited to reflecting the clinical usefulness of a drug for cancer patients. Furthermore, the price difference between drugs approved based on OS and those based on PFS remains unclear. Satoh et al. reported that, in a study of 45 anticancer drugs approved in Japan between 2006 and 2015, there were no correlations between drug prices and PFS, or between drug prices and OS [11]. Considering China, a study involving 58 Chinese anticancer drugs also reported no correlation between clinical benefit and the costs of China’s price-negotiated cancer therapies [12]. Moreover, in a study of 30 anticancer drugs in Italy, the price of these anticancer drugs did not reflect their clinical therapeutic effect [13]. Another study on anticancer drugs approved by the Federal Drug Administration of the United States between 2006 and 2015 reported that the cost of new anticancer drugs has increased rapidly during the past decade; however, the clinical benefit of these drugs has not been proportionate [14]. The existing results generally indicate that there is no correlation between clinical benefit, such as OS or PFS, and drug price. As more and more new anticancer drugs are expected to be launched in the near future, more transparent and reliable pricing methods must be established. Specifically, Japan prefers drug pricing based on clinical usefulness, but the details regarding this are unclear. Therefore, this study investigated the relationship between drug prices and clinical benefits. We also decided to determine whether there is a correlation between clinical benefit, such as OS and PFS, and drug price for recent Japanese anticancer drugs.

The principal objective of this study was to clarify whether OS (the true endpoint) and PFS (the surrogate endpoint) affect anticancer drug prices. The differences between OS and PFS and the relationship between them and drug prices at approval in Japan were investigated.

## 2. Materials and Methods

### 2.1. Selection of Therapeutic Drugs

This study conducted a comprehensive assessment of anticancer drugs approved in Japan between January 2009 and March 2020 in accordance with the authors’ previous report [15]. Only drugs that had been approved as new molecular entities were included, and the analysis was based on the drug prices at the stage of initial approval. The effects of approval—based on additional indications or drug price revisions—were not considered.

We established the definition of anticancer drugs as therapeutic drugs that directly target tumors and malignancies. Therefore, we excluded drugs aimed at treating cancer-related pain, precancerous lesions, drugs for palliative care, diagnostic drugs, and prophylactic drugs for adverse effects.

### 2.2. Data Collection

Drug information was collected from publicly available data. Information regarding applications for approval was mostly obtained from the website of the Pharmaceuticals and Medical Devices Agency of Japan [16]. Information relating to the National Health Insurance (NHI) prices and revisions was obtained from the Central Social Insurance Medical Council’s website [17], the Ministry of Health, Labour and Welfare’s medical insurance website [18], the NHI Drug Price Standards [19], and the NHI Drug Price Standards Quick Reference Tables [20]. Virtually all data were obtained online; however, some information on drug prices were not available online and was procured from books: the NHI Drug Price Standards [19] and the NHI Drug Price Standards Quick Reference Tables [20]. The pharmacological and regulatory characteristics of each anticancer drug were assessed according to our previous work [15,21,22], and an original database was prepared. We conducted this study according to the guideline of Strengthening the Reporting of Observational Studies in Epidemiology (STROBE) [23] for cross-sectional studies.

### 2.3. Data Analysis

The relationships between clinical usefulness and drug prices were analyzed using the following method. First, in relation to clinical usefulness, the pivotal clinical trial that formed the basis for approval of each anticancer drug was identified. The differences between the OS and PFS results in the experimental treatment arm and control treatment arm of the trial were then obtained and calculated as follows:

- Improvement OS (%) = (median OS (days) in experimental treatment arm)/(median OS (days) in control treatment arm);
- Improvement PFS (%) = (median PFS (days)in experimental treatment arm)/(median PFS (days) in control treatment arm);
- ΔOS (days) = (median OS [days] in experimental treatment arm) − (median OS (days) in control treatment arm);
- ΔPFS (days) = (median PFS [days] in experimental treatment arm) − (median PFS (days) in control treatment arm);
- The incremental drug costs of all anticancer drugs until disease progression or death in the experimental treatment arm were defined as the Cost Index (CI), as follows:
- CIpfs (Yen) = drug price/ΔPFS;
- CIos (Yen)= drug price/ΔOS.

### 2.4. Statistical Methods

All statistical analyses were performed using the analytical tools of JMP Pro 15, with a significance level of 5%. JMP Pro 15 was also used to confirm the relationship between continuous variables, and linear regression analysis was performed.

## 3. Results

### 3.1. Background of Approved Drugs

Eighty anticancer drugs were approved in Japan during the 11-year study period. Table 1 shows the regulatory characteristics, drug prices, and primary endpoints of the pivotal clinical trials supporting the approvals for these 80 drugs; Supplementary Table S1 provides further details. In terms of mechanism of action, the most common type of drug was molecular-targeted agents (53.8%), followed by antibody preparations (18.8%). The most common initial indication was solid tumors, followed by hematological cancers. Overall, 53.8% of the drugs were administered orally and 46.3% were injected. The average review period, from application to approval, for 80 drugs was 73.1 (±44.4) days. The maximum review period was 289 days, while the minimum review period was 21 days. The most common endpoint for drug approval was PFS (28 drugs, 35.0%), followed by OS (18 drugs, 22.5%) and RR (13 drugs, 16.3%). The mean (± SD) drug price was JPY 88,416.2 ± 148,974.7, and the median (with quartiles) was JPY 21,694 (JPY 4855–JPY 93,396.8). Drug pricing was based on the cost accounting method and efficacy of similar drugs’ comparison method I, efficacy of similar drugs’ comparison method II, and interspecification adjustment method. Premiums were added for 54 drugs (67.5%), while 56 drugs (70.0%) received corrective premium rates for drug pricing. Of the 80 drugs investigated, 61.3% were applied by a foreign company and 38.8% were by domestic Japanese pharmaceutical companies.

### 3.2. Rates of Increase in OS and PFS Extension and Incremental Drug Costs 

To determine whether there was a difference in drug price, OS and PFS had to be extended by one day, and drug prices and the CI of drugs approved for OS and PFS were compared (Table 2). The results show that the drug prices were significantly higher for PFS than for OS, while CIs were not significantly different between OS and PFS.

### 3.3. Correlation between Improvement Rate of OS and Incremental Drug Costs 

**Figure 1:**
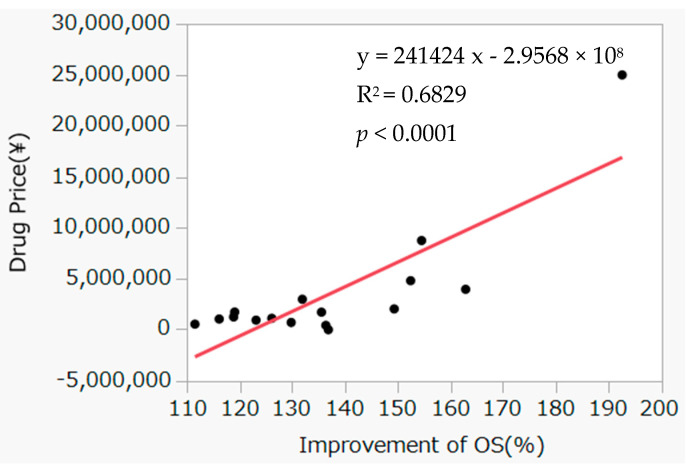
Correlation between improvement rate of overall survival and incremental daily drug price. Red line is the regression line.

**Figure 2:**
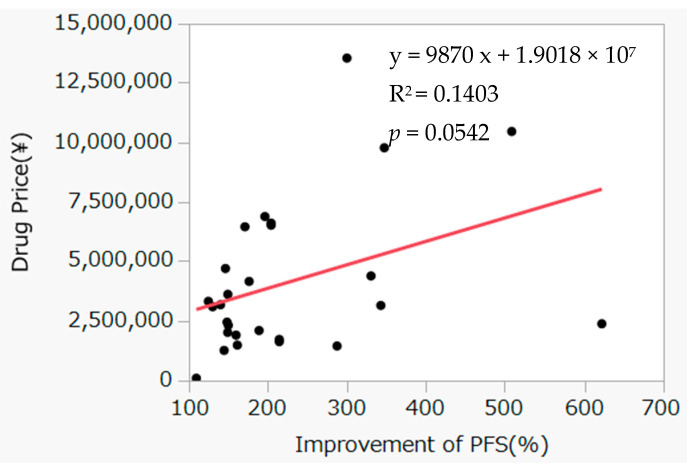
Correlation between improvement rate of progression-free survival and incremental daily drug price. Red line is the regression line.

For 46 drugs, the rates of increase in OS or PFS extension and the associated incremental drug costs (daily drug prices) were compared between drugs approved based on OS and PFS, and a simple calculation was performed for incremental cost effectiveness (Figure 1 and Figure 2). The daily drug price showed a significant correlation with incremental effects on OS but not with incremental effects on PFS.

## 4. Discussion

Drug pricing methods vary across different countries. The method used by the Japanese NHI is unusual and complex [15]. The Japanese Ministry of Health, Labour and Welfare has published drug pricing standards that can be used to set prices for drugs with novel mechanisms of action, drugs that are safer and/or more effective than similar drugs, preparations that have high safety and/or efficacy despite containing similar drugs, and drugs for orphan diseases [19]. The health technology assessment that is accepted by most developed countries is not used for drug price calculation in the Japanese drug pricing system; it is instead used in a supplementary manner at the time of drug price revision [24,25,26]. However, in Japan, the cost effectiveness of high-priced anticancer drugs has recently been the subject of considerable debate between patients, clinicians, and the media [27].

The pivotal study endpoints at the approval stage of anticancer drugs in Japan, as discussed earlier in this study, have been used globally since the revision of the Guidelines for Clinical Evaluation Methods of Anticancer Drugs in 2005 [28]; moreover, there has been a shift in the considered endpoints from tumor contraction to OS extension [6,28]. In the present study, the incremental costs of drugs needed to extend median OS or PFS by one day were calculated using data from the study drug groups and control groups. The incremental drug cost showed no correlation between PFS extension and drug price, whereas a correlation was found between OS extension rate and drug price. In previous studies, no correlation was found between drug price and either PFS or OS [11,12,13,14], differing from results of the present study. One possible cause may be the differences in approval timing of the drugs investigated. Japan’s drug pricing system was radically reformed in 2018, introducing mechanisms for awarding pricing premiums to innovative drugs, such as the novel drug development premium [29]. Consequently, in cases of drugs which demonstrated an extension of OS, the true endpoint, it was assumed that clinical trial results were evaluated, drug pricing premiums were awarded, and correlations between drug prices and clinical usefulness were found. However, this study found no correlation for PFS; this may be because PFS is a surrogate endpoint, and hence, drug pricing premiums were not awarded. This issue must be investigated in future studies.

The objectives of this study were to examine the correlation between OS and PFS and cancer drug prices. To fulfill this objective, we incorporated three items in this study: (1) determining how many times and how often OS and PFS were adopted as the endpoints during the study period of 11 years, and what the characteristics were of the cancer drugs that were approved during these 11 years; (2) investigating whether a difference in drug price was required to extend OS and PFS by one day; and (3) determine whether the rates of increase in OS and PFS correlated with the daily drug price. In summary, this study examined the 80 anticancer drugs approved in Japan between January 2009 and March 2020 and investigated the relationships with drug prices for the 46 drugs that were approved based on OS or PFS endpoints. Investigating the relationships between OS or PFS extension and drug price at the time of listing yielded a correlation for OS but not for PFS.

### Future Issues and Definitions Based on the Present Conclusions

Drug prices and clinical usefulness have not generated much debate in Japan. The prioritized parameter in the development of anticancer agents is OS, the true endpoint [30]. However, evaluation of OS takes considerable time, and recently, the evaluation and approval of numerous drugs have first been achieved based on PFS and/or RR. This is a natural development from the perspective of patients’ access to drugs that are expected to be effective. In the United Kingdom, however, only data for surrogate endpoints of pivotal clinical studies are available, and results based on OS are still not recognized for reimbursement for anticancer drugs [31,32]. Additionally, no correlations were found between RR or PFS and OS [33]. However, a correlation between drug prices and clinical usefulness is essential, and the authors consider a correlation between OS extension and drug price to be important. The present system in Japan awards a premium for the verification of the actual clinical usefulness of a drug, and in 2016, another system was introduced which awarded a premium if efficacy was demonstrated based on the true endpoint after marketing [34]. Most drugs that have been awarded premiums based on these criteria have been only antidiabetic and antihyperlipidemic agents. These systems have received little recognition, even within Japan. The authors suggest that these systems be applied to a greater extent to anticancer drugs that show clinical usefulness based on OS. It is expected that the Japanese people will demand assurance of high clinical usefulness to justify high drug prices.

In conclusion, we investigated the relationship between drug prices and clinical benefits, such as OS and PFS, with anticancer drugs approved from 2009 to 2020 in Japan. We found drug prices were significantly higher for PFS than for OS, while the drug price to prolong one day for PFS or OS did not differ significantly between PFS and OS. We also found a correlation between drug prices and OS but not for PFS.

This study has several limitations. First, it included only anticancer drugs approved based on OS or PFS and not RR or other parameters. Second, it is a retrospective survey based on publicly available information and not a prospective study. Third, multiple anticancer drugs are often administered concomitantly, but this study analyzed incremental drug prices for monotherapy. It is difficult to avoid these limitations by using other analytical model or other parameters in this study.

## Figures and Tables

**Table 1:** Characteristics of oncology approvals between January 2009 and March 2020 in Japan.

Characteristic		Number	Percentage
Compound type	Molecular-targeted drug	43	53.8%
	Antibody	15	18.8%
	Cytotoxic drug	11	13.8%
	Hormonal drug	6	7.5%
	Immunotherapy drug	5	6.3%
	Other	0	0.0%
Indication	Hematologic tumor	30	37.5%
	Solid tumor	50	62.5%
Dosage form	Oral	43	53.8%
	Injection	37	46.3%
Review period (days)	Average (±SD)	73.1 (±44.4)	
	Median (interquartile)	21,694 (4855.0–93,396.8)	
	Maximum	289	
	Minimum	21	
Primary endpoint support approval	OS	18	22.5%
	PFS	28	35.0%
	Response rate	13	16.3%
	Time to Progression	3	3.8%
	Others	18	22.5%
NHI drug price	Average (±SD; Yen)	88,416.2 (±148,974.7)	
	Median (interquartile) (Yen)	21,694 (4855.0–93,396.8)	
	<JPY 1000	2	2.5%
	<JPY 10,000	34	42.5%
	<JPY 100,000	25	31.3%
	<JPY 1,000,000	19	23.8%
	>JPY 1,000,000	0	0.0%
Calculation system for NHI price standard	Cost accounting method	24	30.0%
	Similar efficacy comparison method (I)	51	63.8%
	Similar efficacy comparison method (II)	3	3.8%
	Inter-specification adjustment	2	2.5%
Corrective premium rate	With premium rate	56	70.0%
	None	24	30.0%
Type of pharmaceutical company	Japanese domestic company	31	38.8%
	Foreign company	49	61.3%

NHI: National Health Insurance; OS: overall survival; PFS: progression-free survival.

**Table 2:** Drug price and cost index for overall survival and progression-free survival.

	Overall Survival (*n* = 16)	Progression Free Survival (*n* = 28)	*p* Value
Drug Price (Yen)	208,877	1,372,417	0.0223
Cost Index (Yen)	205,431	64,540	0.2297

## Data Availability

The data presented in this study are available in the article/supplementary materials, further inquiries can be directed to the corresponding author.

## Supplementary Materials

The following supporting information can be downloaded at: https://www.mdpi.com/article/10.3390/curroncol30020137/s1, Table S1: List of oncology drugs approved in Japan from 2009 to 2020.
